# Correspondence

**Published:** 1864-02

**Authors:** John Sheldon

**Affiliations:** Glencoe Mills, N. Y.


GLENCOE MILLS, N. Y., Feb. 1st, 1864.

DR. B. WOOD,—*Sir:* You remember the last time I called on you, that you spoke of filling teeth on the bare nerve with the Plastic Metallic Filling. I have since then tried the experiment in two or three cases, one of which I will mention. On Christmas-day I filled a tooth for a lady (one of the incisors, of which was badly decayed,) and while excavating it, accidentally cut into the pulp cavity until the blood followed the excavator.

I cleansed the cavity with alcohol, and immediately after filled it with the Plastic Metallic Filling on the bare wound. She said that it was a little sore at first, but lasted only for a few days. I have used nothing since then to prevent the sensation of heat, except a little caution in regard to putting the Filling in the bottom of the cavity too warm. Those for whom I have filled teeth with the "Plastic Filling," that have had any filled with gold or tin foil, say that it is easier borne, where the tooth is sensitive. Another reason some have given me why they prefer the "Plastic Filling" is, that the cavity can be filled with it in less time than with either of the above.

Yours truly,

JOHN SHELDON.

